# Aromatic-Turmerone Analogs Protect Dopaminergic Neurons in Midbrain Slice Cultures through Their Neuroprotective Activities

**DOI:** 10.3390/cells10051090

**Published:** 2021-05-03

**Authors:** Yuria Hori, Reiho Tsutsumi, Kento Nasu, Alex Boateng, Yasuhiko Ashikari, Masaharu Sugiura, Makoto Nakajima, Yuki Kurauchi, Akinori Hisatsune, Hiroshi Katsuki, Takahiro Seki

**Affiliations:** 1 Department of Chemico-Pharmacological Sciences, Graduate School of Pharmaceutical Sciences, Kumamoto University, Kumamoto 860-8555, Japan; 192y1010@st.kumamoto-u.ac.jp (Y.H.); lox.mps@gmail.com (R.T.); 217y1013@st.kumamoto-u.ac.jp (K.N.); kurauchy@kumamoto-u.ac.jp (Y.K.); hisatsun@gpo.kumamoto-u.ac.jp (A.H.); hkatsuki@gpo.kumamoto-u.ac.jp (H.K.); 2 Graduate School of Pharmaceutical Sciences, Sojo University, Kumamoto 860-0082, Japan; alexboat77@gmail.com (A.B.); msugiura@ph.sojo-u.ac.jp (M.S.); 3 Department of Organic Chemistry, Graduate School of Pharmaceutical Sciences, Kumamoto University, Kumamoto 860-8555, Japan; y.ashikari@nippon-soda.co.jp (Y.A.); nakajima@gpo.kumamoto-u.ac.jp (M.N.)

**Keywords:** aromatic-turmerone, dopaminergic neurons, microglia, Nrf2

## Abstract

Parkinson’s disease (PD) is a neurodegenerative disorder characterized by the loss of dopaminergic neurons in the substantia nigra. The inflammatory activation of microglia participates in dopaminergic neurodegeneration in PD. Therefore, chemicals that inhibit microglial activation are considered to have therapeutic potential for PD. Aromatic (ar)-turmerone is a main component of turmeric oil extracted from *Curcuma longa* and has anti-inflammatory activity in cultured microglia. The aims of the present study are (1) to investigate whether naturally occurring *S*-enantiomer of ar-turmerone (S-Tur) protects dopaminergic neurons in midbrain slice cultures and (2) to examine ar-turmerone analogs that have higher activities than S-Tur in inhibiting microglial activation and protecting dopaminergic neurons. *R*-enantiomer (R-Tur) and two analogs showed slightly higher anti-inflammatory effects in microglial BV2 cells. S- and R-Tur and these two analogs reversed dopaminergic neurodegeneration triggered by microglial activation in midbrain slice cultures. Unexpectedly, this neuroprotection was independent of the inhibition of microglial activation. Additionally, two analogs more potently inhibited dopaminergic neurodegeneration triggered by a neurotoxin, 1-methyl-4-phenylpyridinium, than S-Tur. Taken together, we identified two ar-turmerone analogs that directly and potently protected dopaminergic neurons. An investigation using dopaminergic neuronal precursor cells suggested the possible involvement of nuclear factor erythroid 2-related factor 2 in this neuroprotection.

## 1. Introduction

Parkinson’s disease (PD) is a neurodegenerative disease characterized by progressive motor symptoms, such as bradykinesia, resting tremor, and rigidity [1,2]. Dopaminergic neurons in the substantia nigra pars compacta (SNpc) of the midbrain selectively and progressively degenerate in patients with PD [1,2,3]. However, it remains unknown how the dopaminergic neurons in the SNpc selectively degenerate in PD. Reactive microglia are frequently accumulated in the SNpc of patients with PD [4,5]. We have previously demonstrated that lipopolysaccharide (LPS) injection into SNpc triggers microglial activation and dopaminergic neurodegeneration in mice [6]. In addition, we established an experimental model of inflammatory degeneration of dopaminergic neurons using midbrain slice cultures that are treated with interferon γ (IFNγ) and LPS to trigger microglial activation [7] and revealed that chemicals that inhibit microglial activation protect dopaminergic neurodegeneration [6,7,8]. Taken together, the inflammatory activation of microglia is one of the key regulators of dopaminergic neurodegeneration in PD.

Aromatic (Ar)-turmerone is one of the main constituents of turmeric oil from *Curcuma longa* [9,10]. Several reports have demonstrated that ar-turmerone has antitumor and anti-inflammatory activities [11,12,13]. Notably, ar-turmerone hampers the LPS- or β-amyloid-triggered inflammatory activation of cultured microglia [14,15]. It also inhibits glial activation and memory impairment induced by chronic and intraperitoneal treatment with LPS [16] and enhances proliferation and differentiation into neurons of neural stem cells [17]. Taken together, ar-turmerone may protect dopaminergic neurons from the inflammatory toxicity of activated microglia in PD models.

Ar-turmerone has an asymmetric carbon and is naturally present as an *S*-enantiomer [18]. It remains unknown whether its *R*-enantiomer and racemic mixture have similar biological activities to those of the *S*-enantiomer. In addition, we have previously synthesized several analogs of ar-turmerones [19,20]. The aims of the present study were (1) to investigate whether ar-turmerone protects dopaminergic neurons in the midbrain slice cultures and (2) to examine ar-turmerone analogs that have higher activities than S-Tur in inhibiting microglial activation and protecting dopaminergic neurons using microglial BV2 cells and midbrain slice cultures. Several ar-turmerone analogs protected dopaminergic neurons without the inhibition of microglial activation. In addition, two analogs activated nuclear factor erythroid 2-related factor 2 (Nrf2) and enhanced the anti-oxidative responses in dopaminergic neuronal precursor cells. We propose the possibility that ar-turmerone analogs directly protect dopaminergic neurons through the activation of Nrf2.

## 2. Materials and Methods

### 2.1. Chemical Synthesis of Ar-Turmerone Analogs

**Figure 1:**
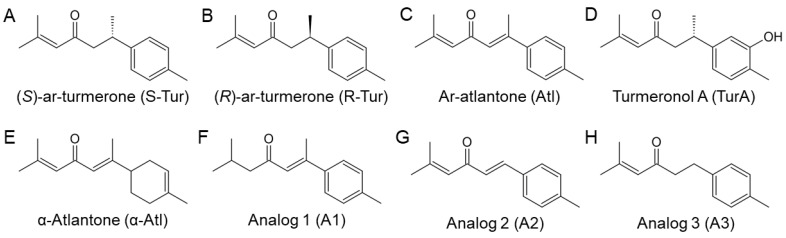
Chemical structures of ar-turmerone analogs. (**A**–**H**) Chemical structures of (S)-ar-turmerone (**A**), (R)-ar-turmerone (**B**), ar-atlantone (**C**), turmeronol A (**D**), α-atlantone (**E**), analog 1 (**F**), analog 2 (**G**), and analog 3 (**H**).

The chemical structures of ar-turmerone analogs are shown in Figure 1A–H. The synthetic procedures of (*S*)-ar-turmerone, (*R*)-ar-turmerone, ar-atlantone, turmeronol A, α-atlantone, and analog 1 have been described previously [19,20]. Analogs 2 and 3 were prepared according to the reported method [20,21]. Detailed methods for preparing analogs 2 and 3 are described in the supplementary materials and are shown in Figure S1. All chemicals were purified to >98% by high-performance liquid chromatography.

### 2.2. Cultivation of Cells and Slices and Drug Treatment

Murine microglial BV2 cells were maintained in Dulbecco’s modified Eagle medium (DMEM, catalog No. 05919, Nissui Pharmaceutical, Tokyo, Japan) containing 10% fetal bovine serum, 100 units/mL penicillin, 100 μg/mL streptomycin, and 2 mM glutamine at 37 °C in a humidified incubator under 5% CO_2_.

For drug treatment, cells were seeded in 35 mm dishes at a density of 4.0 × 10^5^ cells per dish. After 24 h of cultivation, the culture media were replaced with serum-free DMEM. After 3 h of incubation, cells were cultured for 24 h with serum-free DMEM containing 10 ng/mL lipopolysaccharide (LPS, from *Escherichia coli*, serotype 0111:B4, catalog No. L2630, Sigma-Aldrich, St. Louis, MO, USA) and vehicle (0.1% dimethyl sulfoxide (DMSO)) or 20 μM ar-turmerone analogs.

Midbrain slice cultures were prepared, as described in our previous studies [22,23]. Briefly, coronal midbrain slices (350 μm thick) were prepared from 2–3-day-old neonatal Wistar rats (Nihon SLC, Shizuoka, Japan) and placed on microporous membranes (Millicell-CM, catalog No. PICM0RG50, Merck Millipore, Darmstadt, Germany) in six-well plates. A culture medium, consisting of 50% minimum essential medium/HEPES (catalog No. 12360-038, Thermo Fisher Scientific, Waltham, MA, USA), 25% Hanks’ balanced salt solution (catalog No. 24020-117, Themo Fisher Scientific, Waltham, MA, USA), and 25% heat-inactivated horse serum (catalog No. 16050-122, Themo Fisher Scientific) supplemented with 6.5 mg/mL glucose, 2 mM L-glutamine, and 10 U/mL penicillin-G plus 10 μg/mL streptomycin was supplied at a volume of 0.7 mL per well. Slices were cultured at 34 °C in a humidified incubator under 5% CO_2_ for 20 days in vitro (DIV). For immunohistochemistry experiments, the slices were fixed with 4% paraformaldehyde in 0.1 M phosphate buffer containing 4% sucrose at 4 °C for 2.5 h. Culture media samples were collected for nitrite quantification.

Rat interferon γ (IFNγ) (catalog No. 400-20, PeproTech, Cranbury, NJ, USA) and LPS were used to activate the microglia in the slice cultures [23]. At DIV17-20, the midbrain slice cultures were treated for 24 h with IFNγ (50 ng/mL) in serum-free medium, followed by LPS (10 ng/mL) treatment for 72 h. Vehicle (0.1% DMSO) or 20 μM ar-turmerone analogs were treated during stimulation with both IFNγ and LPS. To reduce microglia, the slices were cultured in the presence of 2 μM PLX5622 (Catalog No. 28927, Cayman Chemical, Ann Arbor, MI, USA), an inhibitor of colony-stimulating factor 1 receptor (CSF1R), from 7 DIV. At 19–20 DIV, the slices were treated with 10 μM 1-methyl-4-phenylpyridinium (MPP^+^, catalog No. D048, Sigma-Aldrich) in the presence of 2 μM PLX5622 in serum-free medium. Vehicle (0.1% DMSO) or 20 μM ar-turmerone analogs were simultaneously treated with MPP^+^ for 48 h.

LUHMES cells were purchased from Applied Biological Materials (catalog No. T0284, Richmond, BC, Canada) and cultured, as described in a previous study [24]. Briefly, culture dishes were coated with 50 µg/mL poly-L-ornithine (catalog No. P3655, Sigma-Aldrich) and 1 µg/mL fibronectin (catalog No. 160-49, Nacalai Tesque, Kyoto, Japan). LUHMES cells were cultured in Advanced DMEM/F12 (catalog No. 12634010, Thermo Fisher Scientific) supplemented with 2 mM L-glutamine, 40 ng/mL recombinant basic fibroblast growth factor (catalog No. Z101455, Applied Biological Materials), and N-2 MAX media supplement (catalog No. AR009, R&D Systems, Minneapolis, MN, USA) at 37 °C in a humidified incubator under 5% CO_2_.

For drug treatment, cells were seeded in 35 mm dishes (1.0 × 10^6^ cells/dish) or 8-well glass-bottom chambers (5.0 × 10^4^ cells/dish). After 24 h of cultivation, cells were cultured in medium containing 10 μM MPP^+^ with vehicle (0.1% DMSO) or 20 μM ar-turmerone analogs for 48 h.

### 2.3. Isolation of RNA and Reverse Transcription–Quantitative Polymerase Chain Reaction

Reverse transcription–quantitative polymerase chain reaction (RT-qPCR) was conducted as previously described [25]. After drug treatment for the indicated periods, total RNA was extracted from cultured cells or slices using RNAiso plus (catalog No. 9109, TaKaRa-Bio, Shiga, Japan), according to the manufacturer’s protocol. RT was performed using PrimeScript**^®^** RT Master Mix (Catalog No. RR036B, TaKaRa-Bio). Real-time qPCR was performed using a KAPA SYBR Fast qPCR Kit (catalog No. KAPA Biosystems, Wilmington, MA, USA) on a CFX Connect™ real-time system (Bio-Rad Laboratories, Hercules, CA, USA). The primer sequences are listed in Table 1. The reactions were quantified by selecting the amplification cycle when the PCR product of interest was first detected (the threshold cycle). Data were analyzed with the comparative threshold cycle method. The glyceraldehyde 3-phosphate dehydrogenase (GAPDH) mRNA level was used as the internal control for each sample. The Ct values of GAPDH in all RT-qPCR experiments are shown in Figure S2. The Ct values of GAPDH in each experiment were relatively similar and were not significantly affected by any treatment.

### 2.4. Immunohistochemistry

Cultured slices or cells were fixed with 4% paraformaldehyde in 0.1 M phosphate buffer containing 4% sucrose. Immunohistochemical examinations of midbrain slice cultures were performed, according to the previously described method [22,23]. For permeabilization and blocking, fixed slices were incubated for 1 h at room temperature with PBS containing 0.3% Triton X-100 and 3% normal donkey serum (NDS). Then, slices were incubated overnight at 4 °C with a primary antibody solution in PBS containing 0.3% Triton X-100 and 3% NDS. The primary antibodies used were anti-tyrosine hydroxylase (TH) mouse monoclonal antibody (1:500; catalog No. MAB318, Merck Millipore), anti-ionized calcium-binding adapter protein 1 (Iba1) rabbit polyclonal antibody (1:400; catalog No. 019-19741, FUJIFILM Wako Pure Chemicals, Osaka, Japan), anti-TH rabbit polyclonal antibody (1:500; catalog. No. AB152, Merck Millipore), and anti-inducible nitric oxide synthase (iNOS) mouse monoclonal antibody (1:500; catalog No. 610328, BD Biosciences, San Jose, CA, USA). After three washes with PBS, slices were incubated for 1 h at room temperature in Alexa Fluor 488- or Alexa Fluor 555-conjugated secondary antibodies (1:500, Thermo Fisher Scientific) in PBS containing 0.3% Triton X-100 and 3% NDS.

Immunohistochemical examinations of LUHMES cells were performed, according to the previously described method [26]. Fixed cells were incubated for 10 min at room temperature with PBS containing 0.3% Triton X-100 and 5% normal donkey serum for permeabilization and blocking. After three washes with Tris-buffered saline containing 0.01% Tween 20 (TBS-T), cells were incubated for 1–2 h with TBS-T containing 5% donkey serum and anti-Nrf2 rabbit polyclonal antibody (1:500; catalog No. 16396-1-AP, ProteinTech, Rosemont, IL, USA), followed by incubation for 1 h with TBS-T containing 5% donkey serum, Alexa555-anti-rabbit IgG antibody (1:500; Thermo Fisher Scientific), and 500 ng/mL 4′,6-diamidine-2′-phenylindole dihydrochloride (DAPI, catalog No. D8417, Sigma-Aldrich) for nuclear staining.

Fluorescent images of immunostained slices and cells were obtained using a fluorescence microscope (BIOREVO, Keyence, Osaka, Japan) or a confocal laser microscope (TCS SP5, Leica Biosystems, Nussloch, Germany). The survival of dopaminergic neurons was evaluated by counting TH-positive neurons around regions corresponding to the substantia nigra in the cultured slices. The nuclear localization of Nrf2 was quantified as the percentage of nuclear immunoreactivity in the total immunoreactivity of each cell using the ImageJ software (National Institute of Health, Bethesda, MD, USA).

### 2.5. Nitrite Quantification

The concentration of nitrite in the culture medium reflects the amount of NO released from the cultured slices. Its concentration was determined with a colorimetric Griess assay. The culture media were reacted with an equal volume of Griess reagent (catalog No. G4410, Sigma-Aldrich) for 10 min at room temperature. The amount of diazonium compound was determined based on the absorbance at a wavelength of 560 nm.

### 2.6. Immunoblotting

Cellular lysates of LUHMES cells were analyzed by immunoblotting, according to the previously described method [27]. Cells were lysed with radioimmunoprecipitation assay (RIPA) buffer, followed by sonication. Protein concentrations of the cell lysates were quantified by the bicinchoninic acid assay. Equal amounts of proteins from cell lysates were subjected to SDS-PAGE, followed by immunoblot analyses using anti-Nrf2 rabbit polyclonal antibody (1:1000), anti-kelch-like ECH-associated protein 1 (Keap1) rabbit polyclonal antibody (1:2000; catalog No. 10503-2-AP, ProteinTech), anti-heme oxygenase-1 (HO-1) rabbit polyclonal antibody (1:1000; catalog No. GTX101147, GeneTex, Irvine, CA, USA), anti-NAD(P)H quinone dehydrogenase 1 (NQO1) rabbit polyclonal antibody (1:1000; catalog No. GTX100235, GeneTex), anti-glutamate-cysteine ligase catalytic subunit (GCLC) mouse monoclonal antibody (1:500; catalog No. sc-55536 Santa Cruz Biotechnology, Santa Cruz, CA, USA), anti-glutamate-cysteine ligase modifier subunit (GCLM) rabbit polyclonal antibody (1:1000; catalog No. GTX114075, GeneTex), and anti-β-actin mouse monoclonal antibody (1:2000; catalog No. A2228, Sigma-Aldrich) as primary antibodies. BlueStar Prestained Protein Ladder (catalog No. NE-MWP03, Nippon Genetics, Tokyo, Japan) was used for the molecular weight markers.

### 2.7. Statistical Analyses

The statistical differences of all quantitative data were determined with one-way ANOVA, followed by Tukey’s multiple comparison tests, or Kruskal–Wallis tests, which were followed by Dunn’s multiple comparison tests using the GraphPad Prism 6 software (GraphPad Software, San Diego, CA, USA). Probability values of less than 0.05 were considered significant.

## 3. Results

### 3.1. Effects of Aromatic-Turmerone Analogs on the Inflammatory Activation of Microglial BV2 Cells

**Figure 2:**
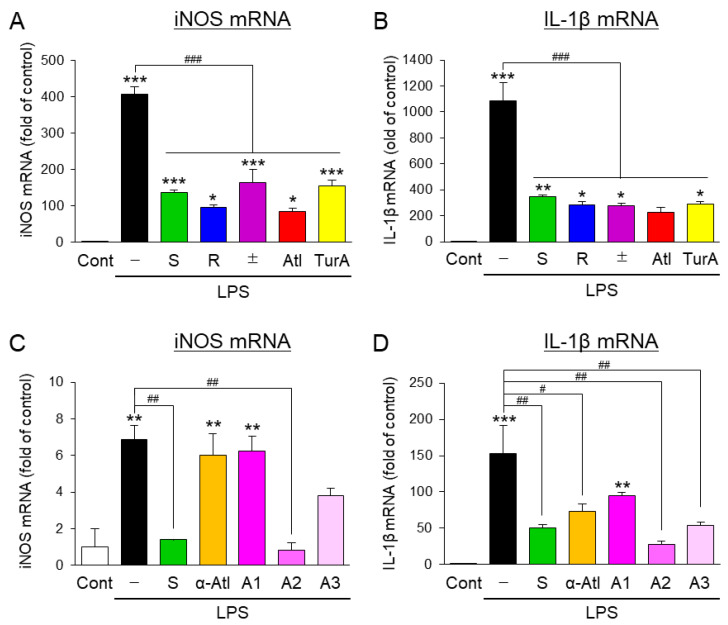
Effects of ar-turmerone analogs on the LPS-induced inflammatory activation of microglial BV2 cells. The mRNA levels of iNOS (**A**,**C**) and IL-1β (**B**,**D**) in BV2 cells were quantified with RT-qPCR. BV2 cells were treated with 10 ng/mL LPS in the presence or absence of 20 μM ar-turmerone analogs (S-Tur, R-Tur, ±-Tur, Atl, and TurA in **A**, **B**, S-Tur, α-Atl, A1, A2, and A3 in **C**, **D**) for 24 h. The data are presented as the mean ± SEM of three independent samples. * *p* < 0.05, ** *p* < 0.01, *** *p* < 0.001 vs. control, # *p* < 0.05, ## *p* < 0.01, ### *p* < 0.001 (n = 3, one-way ANOVA, followed by a post hoc Tukey test).

The chemical structures of the naturally occurring S-enantiomer of ar-turmerone ((S)-ar-turmerone (S-Tur)) and its analogs are shown in Figure 1. We first compared the anti-inflammatory effects of S-Tur, an R-enantiomer of ar-turmerone ((R)-ar-turmerone (R-Tur)), a racemic mixture of S-Tur and R-Tur ((±)-ar-turmerone (±-Tur)), ar-atlantone (Atl), and turmeronol A (TurA) (Figure 1A–D) on the LPS-triggered inflammatory activation of BV2 cells. All analogs were treated at 20 μM because S-Tur prominently inhibited LPS-induced microglial activation at this concentration in a previous study [14].BV2 cells were concomitantly treated with each analog and LPS (10 ng/mL) for 24 h. All five compounds significantly inhibited the mRNA elevation of iNOS and IL-1β, which are inflammatory factors and are upregulated by LPS treatment (Figure 2A,B). Among the four analogs of S-Tur, R-Tur, and Atl inhibited the LPS-induced elevation of iNOS mRNA more potently than S-Tur (Figure 2A), although all analogs similarly hampered the upregulation of IL-1β mRNA (Figure 2A). To obtain better derivatives, we synthesized four different analogs from Atl: α-atlantone (α-Atl) and three unnamed compounds (analog 1–3, A1–A3) (Figure 1E-H). Among these four analogs, one analog (A2) significantly inhibited the LPS-triggered elevation of iNOS mRNA (Figure 2C). Regarding IL-1β mRNA, α-Atl, A2, and A3 significantly prevented its upreglulation (Figure 2D). A2 showed a stronger inhibitory effect than that of S-Tur on the elevation of both inflammatory factors in BV2 cells.

### 3.2. Effects of Aromatic-Turmerone Analogs on Dopaminergic Neurodegeneration Triggered by Microglial Activation in Midbrain Slice Cultures

**Figure 3:**
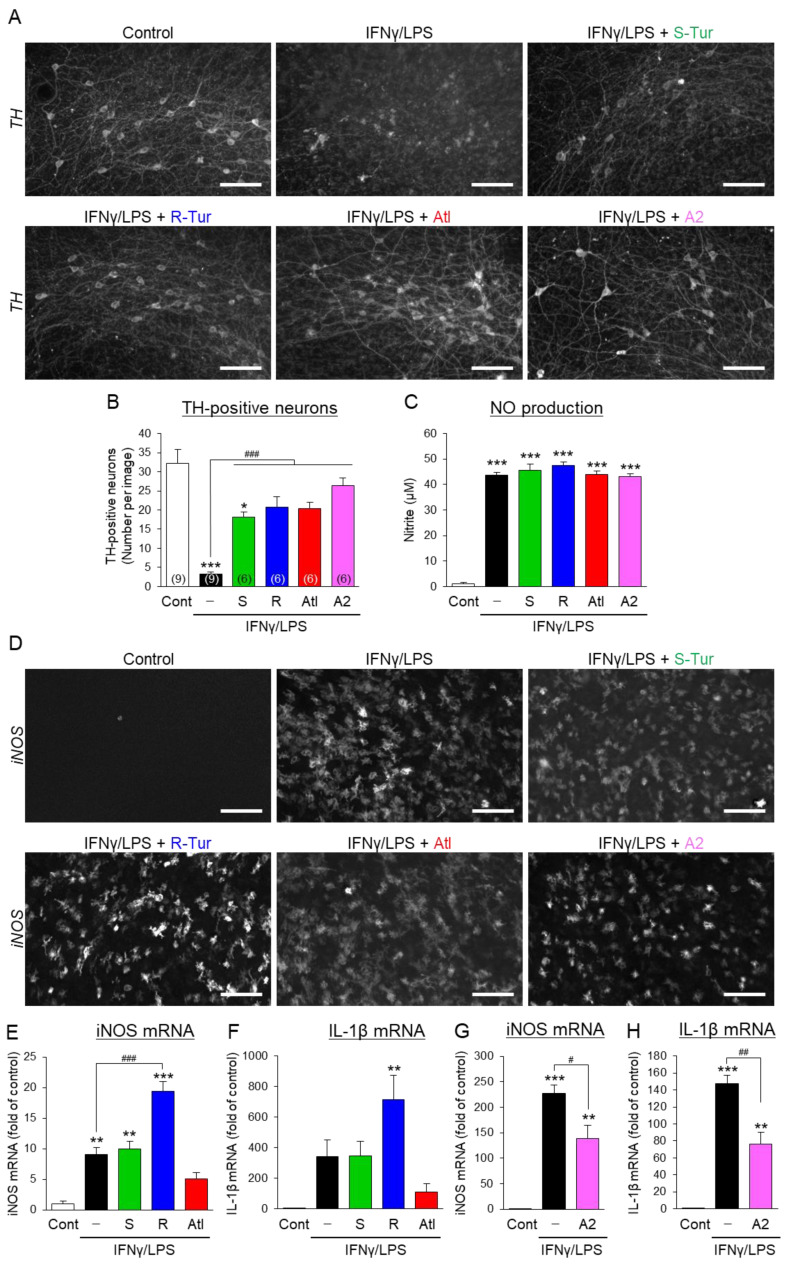
Effects of ar-turmerone analogs on dopaminergic neurodegeneration and microglial activation triggered by the treatment of IFNγ/LPS in midbrain slice cultures. (**A**) Representative images of TH immunoreactivity in midbrain slice cultures under control conditions, treatment with 50 ng/mL IFNγ and 10 ng/mL LPS (IFNγ/LPS) alone, and cotreatment with IFNγ/LPS and 20 μM ar-turmerone analogs. Scale bars, 100 μm. (**B**) Quantitative analyses of TH-positive neurons in cultured midbrain slices. Data are presented as the mean ± SEM of 6–9 independent samples. Numbers in parentheses indicate biological replicates of each group. * *p* < 0.05, *** *p* < 0.001 vs. control, ### *p* < 0.001 (n = 6–9, one-way ANOVA, followed by a post hoc Tukey test). (**C**) Nitrite concentrations in the culture media from midbrain slice cultures. The nitrite concentration represents the amount of released NO from cultured slices. Data are presented as the mean ± SEM of 6–9 independent samples. *** *p* < 0.001 vs. control (n = 3, one-way ANOVA, followed by a post hoc Tukey test). (**D**) Representative images of iNOS immunoreactivity in midbrain slice cultures. Scale bars, 100 μm. (**E**–**H**) The mRNA levels of iNOS (**E**,**G**) and IL-1β (**F**,**H**) in cultured slices were quantified with RT-qPCR. Data in E–H are presented as the mean ± SEM of three independent samples. ** *p* < 0.01, *** *p* < 0.001 vs. control, # *p* < 0.05, ## *p* < 0.01, ### *p* < 0.001 (n = 3, one-way ANOVA, followed by a post hoc Tukey test).

Next, we investigated the effect of S-Tur and three analogs (R-Tur, Atl, and A2) that showed stronger anti-inflammatory effects than those of S-Tur in BV2 cells on dopaminergic neurodegeneration triggered by sequential treatments of IFNγ and LPS in midbrain slice cultures from rats [7]. These four compounds significantly prevented the loss of tyrosine hydroxylase (TH)-positive dopaminergic neurons (Figure 3A,B). Nitric oxide (NO) is involved in the inflammatory degeneration of dopaminergic neurons in the midbrain slice cultures [7]. However, none of these compounds altered the elevation of nitrites, a metabolite of NO, in the culture media (Figure 3C). In addition, none of these compounds affected the elevated immunoreactivity of iNOS in the midbrain slice cultures (Figure 3D). Furthermore, S-Tur and R-Tur did not inhibit the elevation of iNOS and IL-1β mRNA levels, while Atl prevented the significant elevation of iNOS mRNA triggered by IFNγ/LPS (Figure 3E,F). A2 significantly inhibited the elevation of iNOS and IL-1β mRNAs in cultured midbrain slices (Figure 3G,H). However, the inhibitory effects of A2 were around 50% and were smaller than those in BV2 cells (more than 80% inhibition, Figure 2C,D). These findings indicate that ar-turmerone analogs prevent dopaminergic neurodegeneration independently of the inhibition of NO production, although Atl and A2 partly inhibit microglial activation by IFNγ/LPS in cultured midbrain slices.

### 3.3. Effects of Aromatic-Turmerone Analogs on Dopaminergic Neurodegeneration Triggered by 1-Methyl-4-Phenylpyridinium in Midbrain Slice Cultures

**Figure 4:**
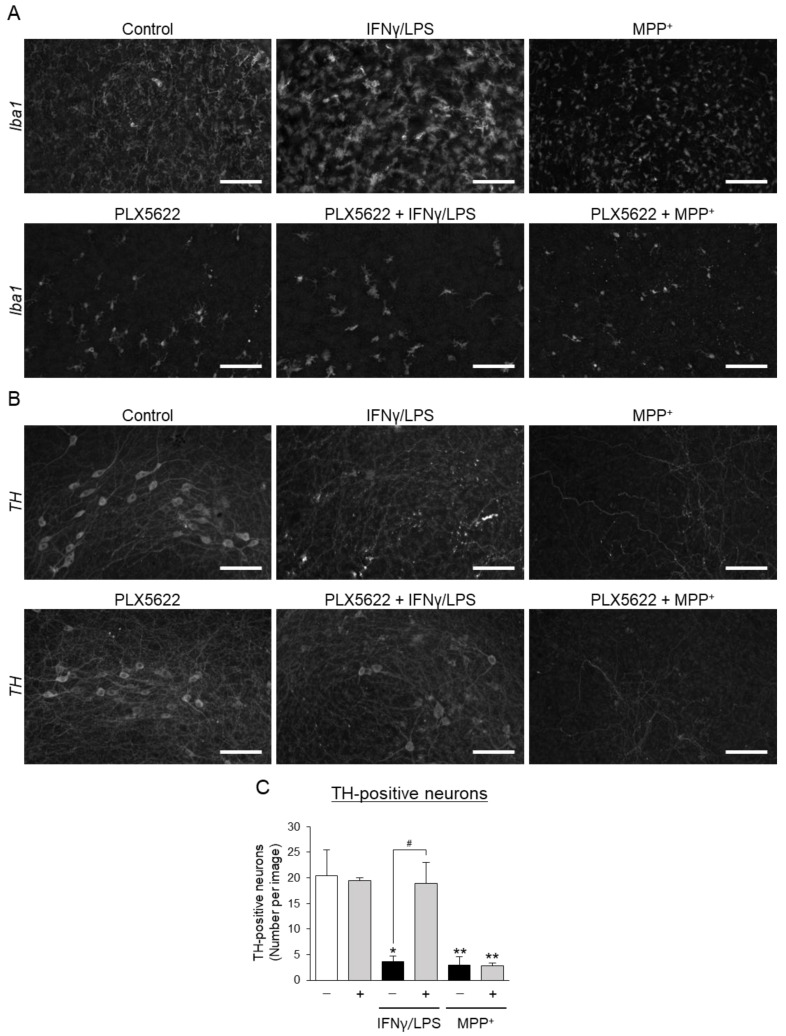
Effect of microglial reduction on dopaminergic neurodegeneration in midbrain slice cultures. (**A**,**B**) Representative images of Iba1 (**A**) and TH (**B**) immunoreactivity in midbrain slice cultures. Slice cultures were maintained in the absence (upper panels) or presence (lower panels) of 2 μM PLX5622, which reduces microglia, for 13 days (DIV7-20). Slices were cultured under the control condition (left), treated with IFNγ/LPS (center), or treated with 10 μM MPP^+^ (right). Scale bars, 100 μm. (**C**) Quantitative analyses of TH-positive neurons in midbrain slice cultures. Data are presented as the mean ± SEM of three independent samples. * *p* < 0.05, ** *p* < 0.01 vs. control, # *p* < 0.05 (n = 3, one-way ANOVA, followed by a post hoc Tukey test).

From the findings in Figure 3, we hypothesized that ar-turmerone analogs directly act on and protect dopaminergic neurons in midbrain slice cultures. To validate this hypothesis, we investigated the effects of these compounds on dopaminergic neurodegeneration triggered by 1-methyl-4-phenylpyridinium (MPP^+^), a neurotoxin that is specific for dopaminergic neurons and frequently used to establish PD models [2]. However, previous studies have revealed that microglial activation is also involved in the neurotoxicity of MPP^+^ [28,29]. To exclude the influence of microglia, we chronically treated midbrain slice cultures with PLX5622, an inhibitor of the colony-stimulating factor 1 receptor (CSF1R), to reduce microglia [30] before treatment with MPP^+^. Although the long-term treatment of 2 μM PLX5622 did not completely deplete microglia, it fully reversed the dopaminergic neurodegeneration triggered by IFNγ/LPS in midbrain slice cultures, suggesting that this reduction in microglia by PLX5622 is enough to eliminate the toxic effect of microglial activation on dopaminergic neurons (Figure 4). PLX5622 did not affect the loss of dopaminergic neurons triggered by MPP^+^ in midbrain slice cultures (Figure 4), excluding the involvement of microglial activation in MPP^+^ toxicity to dopaminergic neurons.

**Figure 5:**
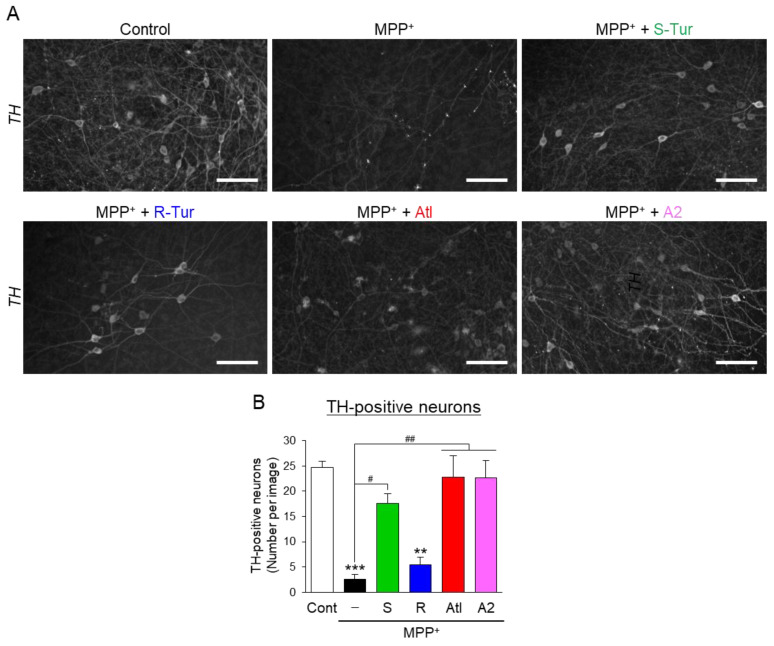
Effects of ar-turmerone analogs on microglial activation triggered by treatment with MPP^+^ in midbrain slice cultures under microglial reduction. (**A**) Representative images of TH immunoreactivity in PLX5622-treated midbrain slice cultures under control conditions, treatment with MPP^+^ alone, and cotreatment with MPP^+^ and 20 μM ar-turmerone analogs. Scale bars, 100 μm. (**B**) Quantitative analyses of TH-positive neurons in midbrain slice cultures. Data are presented as the mean ± SEM of three independent samples. ** *p* < 0.01, *** *p* < 0.001 vs. control, # *p* < 0.05, ## *p* < 0.01 (n = 3, one-way ANOVA, followed by a post hoc Tukey test).

PLX5622-pretreated midbrain slice cultures were simultaneously treated with ar-turmerone analogs and MPP^+^. S-Tur significantly inhibited the loss of dopaminergic neurons triggered by MPP^+^, although its enantiomer, R-Tur, did not affect dopaminergic neurodegeneration by MPP^+^ (Figure 5). Atl and A2 markedly reversed dopaminergic neurodegeneration. These findings indicate that S-Tur and its two analogs directly protect dopaminergic neurons in midbrain slice cultures.

### 3.4. Effects of Aromatic-Turmerone Analogs on Anti-Oxidative Responses in Dopaminergic Neuronal Precursor Cells

**Figure 6:**
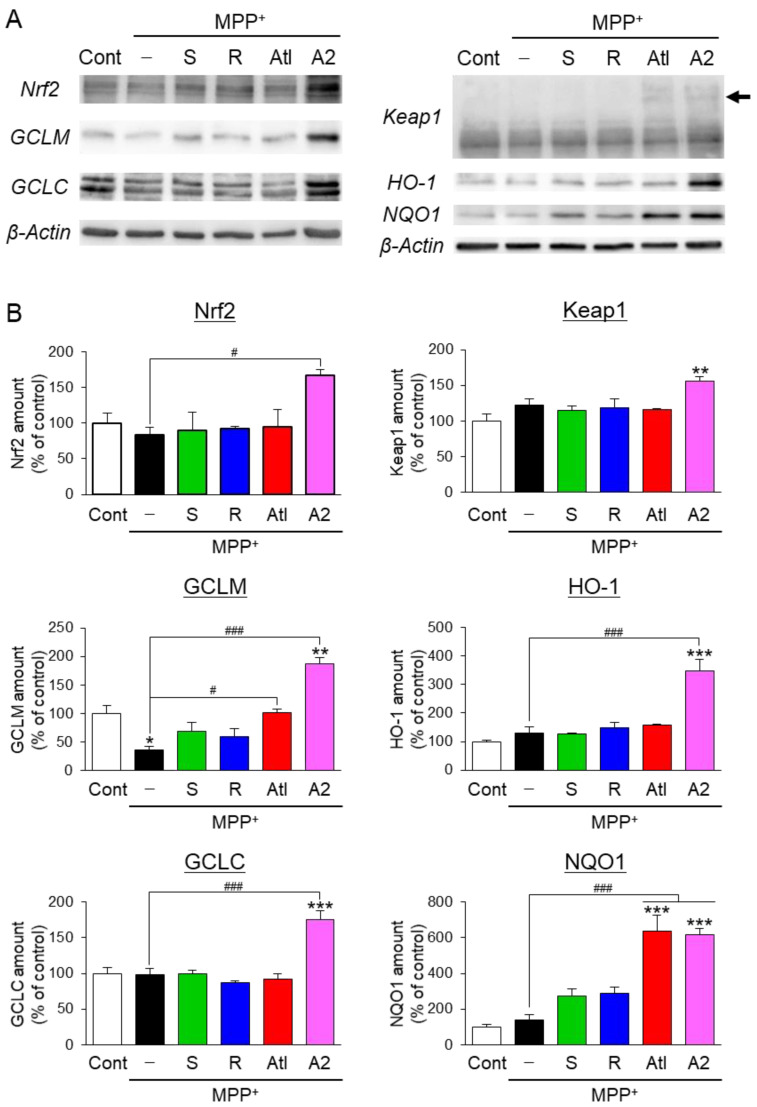
Effects of ar-turmerone analogs on Nrf2-related proteins in LUHMES cells. (**A**) Representative immunoblot images of Nrf2, GCLM, GCLC, Keap1, HO-1, NQO1, and β-actin in LUHMES cells under control conditions, treated with MPP^+^ (10 μM), and cotreated with MPP^+^ and ar-turmerone analogs (20 μM). Whole images of immunoblotting are presented in Figure S3. The arrow in the Keap1 blot indicates high-molecular-weight bands detected in cells treated with Atl and A2. (**B**) Quantitative analyses of immunoreactive bands of Nrf2, GCLM, GCLC, Keap1, HO-1, and NQO1. The band intensities were normalized with the immunoreactivity of β-actin as an internal control. Data are presented as the mean ± SEM of three independent samples. * *p* < 0.05, ** *p* < 0.01, *** *p* < 0.001 vs. control, # *p* < 0.05, ### *p* < 0.001 (n = 3, one-way ANOVA, followed by a post hoc Tukey test).

**Figure 7:**
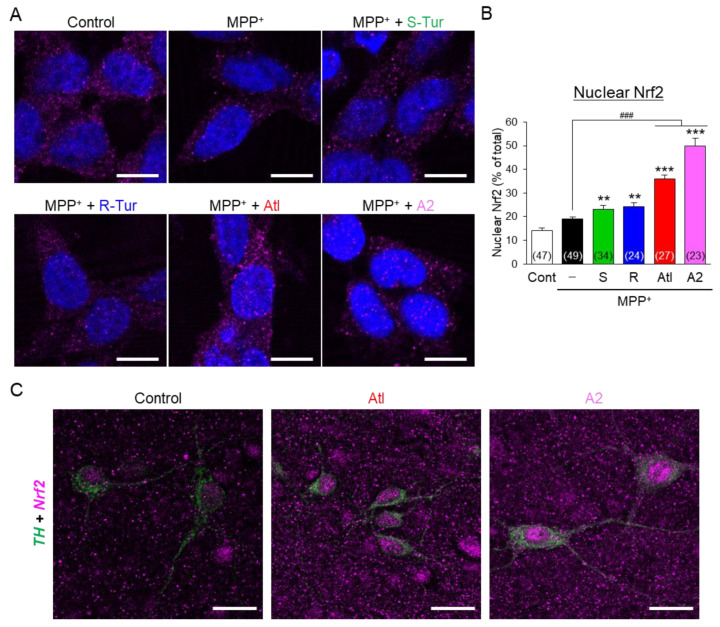
Effects of ar-turmerone analogs on the nuclear expression of Nrf2. (**A**) Representative images of Nrf2 immunoreactivity (magenta) and nuclear staining with DAPI (blue) in LUHMES cells under control conditions, treated with MPP^+^ (10 μM), and cotreated with MPP^+^ and ar-turmerone analogs (20 μM). Scale bars, 10 μm. (**B**) Quantitative analysis of nuclear localization of Nrf2. The percentage of the nuclear Nrf2 in the total Nrf2 represents nuclear localization of Nrf2. Data are presented as mean ± SEM of 23–49 independent cells. Numbers in parentheses indicate biological replicates of each group. ** *p* < 0.01, *** *p* < 0.001 vs. control, ### *p* < 0.001 (n = 23–49, Dunn’s multiple comparisons test). (**C**) Representative images of TH (green) and Nrf2 (magenta) immunoreactivity in midbrain slice cultures under control conditions and treated with Atl or A2 (20 μM). Scale bars, 20 μm.

To elucidate the mechanism of how ar-turmerone analogs directly protect dopaminergic neurons, we used the immortalized dopaminergic neuronal precursor cells, Lund human mesencephalic (LUHMES) cells [24], because the population of dopaminergic neurons is small among all cells in midbrain slice cultures. A previous report demonstrated that ar-turmerone (S-Tur) activates nuclear factor erythroid 2-related factor 2 (Nrf2) in BV2 cells [14]. Activation of Nrf2 upregulates various anti-oxidative proteins, including heme oxidase-1 (HO-1) and NAD(P)H:quinone oxidoreductase 1 (NQO1), and increases glutathione (GSH) through the upregulation of glutamate-cysteine ligase (GCL), a rate-limiting enzyme for GSH synthesis [31]. GCL consists of catalytic (GCLC) and modifier (GCLM) subunits. As MPP^+^ inhibits mitochondrial complex I and increases mitochondrial reactive oxygen species (ROS) in dopaminergic neurons [2], anti-oxidative proteins and GSH would protect dopaminergic neurons from MPP^+^ toxicity. Therefore, we investigated the effects of ar-turmerone analogs on Nrf2 activation. LUHMES cells were treated with 10 μM MPP^+^ in the presence or absence of 20 μM ar-turmerone analogs for 48 h. Immunoblot analyses revealed that co-treatment of A2 and MPP^+^ significantly increased the amount of Nrf2 in LUHMES cells compared with MPP^+^ alone (Figure 6). In addition, immunostaining experiments revealed that Atl and A2 significantly enhanced the nuclear translation of Nrf2 compared with MPP^+^ alone (Figure 7A,B). Furthermore, Atl and A2 prominently increased nuclear Nrf2 in TH-positive dopaminergic neurons in midbrain slice cultures (Figure 7C).

Under normal conditions, Kelch-like ECH-associated protein 1 (Keap1) interacts with Nrf2 in the cytoplasm and negatively regulates the expression of Nrf2 via the ubiquitination and proteasomal degradation of Nrf2 [31]. Keap1 acts as a sensor protein of oxidative and electrophilic stress [32]. In stress conditions, Nrf2 is released from Keap1, increased, and translocated to nuclei, leading to the upregulation of anti-oxidative proteins, as described above. Although the cotreatment of MPP^+^ and A2 significantly increased the amount of Keap1, none of the analogs significantly altered the amount of Keap1, compared with MPP^+^ alone (Figure 6). Interestingly, Atl and A2 triggered slight bandshifts of Keap1 to higher molecular weights (Figure 6A, arrow). A2 significantly increased the expression of GCLM, GCLC, HO-1, and NQO1 compared with MPP^+^ alone (Figure 6). Atl also significantly increased GCLM and NQO1 (Figure 6). These findings indicate that Atl and A2 activate Nrf2 and upregulate anti-oxidative proteins in LUHMES cells.

## 4. Discussion

Consistent with the previous report [14], S-Tur, a naturally occurring ar-turmerone, and several ar-turmerone analogs inhibited the LPS-triggered activation of microglial BV2 cells (Figure 2). As we expected, these analogs significantly inhibited the inflammatory neurodegeneration of dopaminergic neurons in midbrain slice cultures (Figure 3A,B). However, these analogs, unexpectedly, did not inhibit the production of NO (Figure 3C), which participates in the inflammatory neurodegeneration of dopaminergic neurons in midbrain slice cultures [7], or the elevation of iNOS protein (Figure 3D). In addition, these analogs did not sufficiently prevent microglial activation triggered by IFNγ/LPS in midbrain slice cultures (Figure 3E–H). These inconsistent effects of ar-turmerone analogs between BV2 cells and slice cultures may be derived from the difference in culture conditions. Only microglial cells were present in the cultures of BV2 cells and primary microglia [14]. In contrast, neurons, and other glial cells (astrocytes and oligodendrocytes) also existed in the midbrain slice cultures. Ar-turmerone could affect these cells as well as the microglia. Interactions among different types of cells would counteract the anti-inflammatory effect of ar-turmerone analogs on microglia.

Although Atl and A2 prevented the upregulation of iNOS mRNA (Figure 3E,G), these compounds did not affect NO production and iNOS protein expression (Figure 3C,D). We assessed the protein and mRNA expression of iNOS in slice cultures at the endpoint of the treatment (96 h after drug treatment). As changes in mRNA expression precede the changes in protein expression, iNOS protein expression at the endpoint of the treatment should be reflected by iNOS mRNA expression at an earlier time point. Therefore, Atl and A2 might not affect the early changes in iNOS expression, leading to a lack of an influence on NO production and iNOS protein expression. This finding strongly suggests that ar-turmerone analogs directly act on dopaminergic neurons and protect them from toxic inflammation. Indeed, S-Tur, Atl, and A2 significantly inhibited the MPP^+^-triggered degeneration of dopaminergic neurons in microglia-reduced slice cultures (Figure 5).

In contrast, R-Tur did not ameliorate MPP^+^ toxicity, suggesting that the neuroprotective effect of R-Tur is mediated by microglia. However, R-Tur aggravated the microglial activation triggered by IFNγ/LPS (Figure 3D,E). How does it protect dopaminergic neurons? We have previously demonstrated that an activator of peroxisome proliferator-activated receptor γ (PPARγ) triggers the anti-inflammatory activation of microglia without inhibiting inflammatory activation and protects dopaminergic neurons in the midbrain slice cultures [33]. Therefore, one possibility is that R-Tur protects dopaminergic neurons via the induction of anti-inflammatory activation of microglia. However, R-Tur did not affect arginase-1 and interleukin-10 mRNA levels (Figure S4), markers of microglial anti-inflammatory activation. Another possibility is that R-Tur prevents the release of toxic exosomes from inflammatory microglia. We recently revealed that exosomes from activated microglia participate in the inflammatory degeneration of dopaminergic neurons in midbrain slice cultures [23]. In addition, an inhibitor of neutral sphingomyelinase 2 protects dopaminergic neurons from toxic inflammation via the inhibition of exosome release without affecting the microglial activation [23]. Further studies are necessary in order to elucidate how R-Tur affects microglia and protects dopaminergic neurons.

MPP^+^ toxicity is mediated by mitochondrial dysfunction and ROS production in dopaminergic neurons [2]. Therefore, Nrf2-mediated elevation of anti-oxidative proteins would contribute to the neuroprotective effects of Atl and A2, in the present study. Indeed, several studies have revealed that the activation of Nrf2 protects dopaminergic neurons from MPP^+^ neurotoxicity [34,35]. Although a previous study demonstrated that ar-turmerone (S-Tur) increases Nrf2 protein expression in BV2 cells [14], S-Tur did not increase Nrf2 in LUHMES cells (Figure 6). The different time schedules would cause the divergence in the effects of S-Tur on Nrf2 expression between these two cells. Nrf2 was upregulated within 8 h in BV2 cells in the previous study [14]. In contrast, we evaluated the Nrf2 expression after 48 h of treatment in LUHMES cells. As S-Tur induced a slight increase in nuclear translocation of Nrf2 in LUHMES cells (Figure 7A, B), it is possible that S-Tur increased Nrf2 expression at an earlier point. Indeed, 8 h of treatment of S-Tur significantly increased Nrf2 in the presence of MPP^+^ in LUHMES cells (Figure S5). Taken together, the early activation of Nrf2 might participate in the neuroprotective effect of S-Tur.

Keap1 is a cysteine-rich protein that acts as a sensor protein against oxidative and electrophilic stresses [32,36]. Electrophilic compounds interact with the cysteine residues of Keap1 and interrupt the binding of Keap1 to Nrf2, thereby activating Nrf2 [36]. Several electrophilic compounds trigger the ubiquitination of Keap1 with a bandshift to a higher molecular weight in immunoblotting [37], similarly to our immunoblot findings from lysates of LUHMES cells treated with Atl and A2 (Figure 6A, arrow). This finding strongly suggests that Atl and A2 are more electrophilic than S-Tur, bind to cysteine residues of Keap1, activate Nrf2, and increase anti-oxidative proteins and GSH, leading to the protection of dopaminergic neurons.

**Figure 8:**
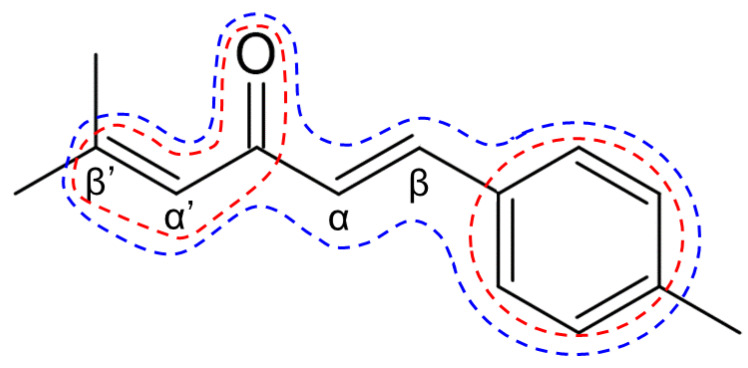
Structure-activity relationship of ar-turmerone analogs. The chemical structure of A2 with indications of α-, β-, α’-, and β’-carbons from the carbonyl base. Red and blue lines represent the regions involved in the anti-inflammation in BV2 cells and Nrf2 activation in LUHMES cells, respectively.

Based on the structure of A2 (Figure 8), we discuss the structure-activity relationship of ar-turmerone analogs. Among the analogs used in the present study, only A1 did not have an anti-inflammatory effect on LPS-activated BV2 cells (Figure 2C,D). Compared with other analogs, A1 lacks an alkene bond between α’- and β’-carbons (Figure 1). An enone structure (a ketone conjugated with an alkene) is reported to be essential for inhibiting nuclear factor-κB and mitogen-activated protein kinase pathways [38,39], which are involved in the inflammatory activation of BV2 cells [14,15]. Although A1 has an enone structure with an alkene between α- and β-carbons, an enone structure with an alkene between α’- and β’-carbons would be crucial for the anti-inflammatory effect on BV2 cells in ar-turmerone analogs (Figure 8, red line). In addition, α-Atl showed a weak anti-inflammatory effect on BV2 cells (Figure 2C,D). As only α-Atl does not have an aromatic ring conjugated to β carbon, this structure is also crucial for the anti-inflammatory effect on BV2 cells (Figure 8, red line).

With regard to the activation of Nrf2, Atl and A2 commonly have dienone structures, but S-Tur and R-Tur do not (Figure 1). As electrons are delocalized between the dienone structure and the aromatic ring in Atl and A2, β- and β’-carbons are highly electrophilic [40,41]. As described above, electrophilic compounds can interact with the cysteine residues of Keap1 and activate Nrf2 [36]. Indeed, among the various derivatives of curcumin, another biological component of *Curcuma longa* [10], compounds that have dienone structures conjugated with aromatic rings more potently activate Nrf2 than curcumin [42]. Therefore, the dienone structure conjugated with an aromatic ring is related to the activation of Nrf2 (Figure 8, blue line). A2 is more potent in the activation of Nrf2 than Atl (Figure 6,7). The difference between these two compounds is the methyl group bound to the β-carbon (Figure 1). It is speculated that the electrophilic β-carbon of Atl and A2 mainly interacts with cysteine residues of Keap1 and that this interaction is structurally attenuated by the methyl group attached to the β-carbon of Atl. The addition of the highly electron-donating groups attached to the aromatic ring of A2 may stabilize the transition state of the reaction with nucleophilic molecules [20,43,44] and may activate Nrf2 more potently.

## 5. Conclusions

In the present study, we first revealed that ar-turmerone and its analogs inhibit dopaminergic neurodegeneration in midbrain slice cultures, probably through the activation of Nrf2 in dopaminergic neurons. This direct neuroprotective effect is supported by the recent finding that ar-turmerone inhibits apoptosis of primary cultured cerebellar granule cells [45]. Among the analogs, A2 has both anti-inflammatory and neuroprotective activities. Therefore, A2 might be a potential candidate for treating PD. As oral administration of ar-turmerone shows anti-depressive activity in mice [46], A2 would also be orally available and penetrate the blood-brain barrier. Further studies are expected to evaluate the in vivo efficacy of A2 on PD model animals.

## Figures and Tables

**Table 1:** Primer sequences used in the RT-PCR analyses.

Gene Name	Forward Primer (5′→3′)	Reverse Primer (5′→3′)
iNOS (mouse, rat)	TGCTTTGTGCGAAGTGTCAGT	CGGACCATCTCCTGCATTTCT
IL-1β (mouse)	TGAAGGGCTGCTTCCAAACC	TGTCCATTGAGGTGGAGAG
IL-1β (rat)	AAAGAAGAAGATGGAAAAGCGGTT	GGAACTGTGCAGACTCAAACTC
IL-10 (rat)	AAAGCAAGGCAGTGGAGCAG	TCAAACTCATTCATGGCCTTGT
Arginase-1 (rat)	CCAGTATTCACCCCGGCTAC	ACAAGACAAGGTCAACGCCA
GAPDH (mouse, rat)	ACCATCTTCCAGGAGCGAGA	CAGTCTTCTGGGTGGCAGTG

## Data Availability

The data presented in this study are available on request from the corresponding author.

## Supplementary Materials

The following are available online at https://www.mdpi.com/article/10.3390/cells10051090/s1, Figure S1: Schematic illustration of the synthetic procedures of analog 2 and analog 3, Figure S2: Ct values of GAPDH in all RT-qPCR experiments, Figure S3: Whole images of immunoblot experiments in Figure 6, Figure S4: The effect of R-Tur on the markers of anti-inflammatory activation of microglia in midbrain slice cultures, Figure S5: Effect of 8 h of treatment with MPP^+^ and S-Tur on Nrf2 expression in LUHMES cells.
