# Supplementary material for: Aromatic-Turmerone Analogs Protect Dopaminergic Neurons in Midbrain Slice Cultures through Their Neuroprotective Activities
Source: Cells. 2021 May 3;10(5):1090. doi: 10.3390/cells10051090 (PMC8147616; doi:10.3390/cells10051090)
Supplement: Supplementary file 1 [file cells-10-01090-s001.zip › cells-1163962-supplementary.pdf]

Supplementary methods

Preparation of Analog 2 (A2)

A solution of NaOH (120 mg, 3 mmol) in H_2O (1.2 mL) was added to a solution of mesityl oxide (2.0 mmol) in EtOH (0.8 mL). After stirring at rt for 5 min, *p*-tolualdehyde (2.0 mmol) was added to the mixture. The mixture was stirred at rt for 24 h and extracted with Et_2O (3 × 20 mL). The combined organic layers were dried over anhydrous Na_2SO_4, filtered, and concentrated under vacuum. The residue was purified by column chromatography on silica gel (Hexane/AcOEt = 10:1) to give A2 (210 mg, 52% yield).

(*E*)-5-Methyl-1-(*p*-tolyl)hexa-1,4-dien-3-one (A2)

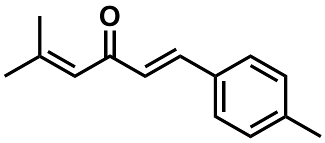

TLC: R_f 0.20 (Hexane/AcOEt = 30:1, stained blue green with phosphomolybdic acid). ^1H NMR (500 MHz, $CDCl_3$) δ 1.97 (s, 3H), 2.22 (s, 3H), 2.37 (s, 3H), 6.34 (s, 1H), 6.75 (d, J = 16.0 Hz, 1H), 7.19 (d, J = 8.0 Hz, 2H), 7.45 (d, J = 8.0 Hz, 2H), 7.55 (d, J = 16.0 Hz, 1H).

Preparation of Analog 3 (A3)

Under argon atmosphere, $HSiCl_3$ in dichloromethane (2.9 M, 0.069 mL, 0.2 mmol) was added to a solution of 1,8-bis(diphenylphosphinyl)naphthalene (5.3 mg, 0.01 mmol), 2,6-lutidine (11.6 µL, 0.1 mmol), A2 (20.3 mg, 0.1 mmol) in CH_2Cl_2 (0.4 mL) at 0 °C. After being stirred at 0 °C for 24 h, the reaction was quenched with sat. $NaHCO_3$. The mixture was stirred for 30 min and filtered through a Celite pad. The filtrate was extracted with CH_2Cl_2 (3 × 10 mL). The combined organic layers were dried over anhydrous $MgSO_4$, filtered, and concentrated under vacuum. The residue was purified by PTLC on silica gel (Hexane/AcOEt = 30:1) to give A3 (16.4 mg, 81% yield).

5-Methyl-1-(*p*-tolyl)hexa-4-en-3-one (A3)

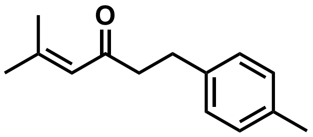

TLC: R_f 0.33 (Hexane/AcOEt = 30:1, stained black with phosphomolybdic acid). ^1H NMR (500 MHz, $CDCl_3$) δ 1.87 (s, 3H), 2.15 (d, J = 1.1 Hz, 3H), 2.31 (s, 3H), 2.71 (t, J = 7.7 Hz, 2H), 2.87 (t, J = 7.7 Hz, 2H), 6.06 (s, 1H), 7.08 (s, 4H).

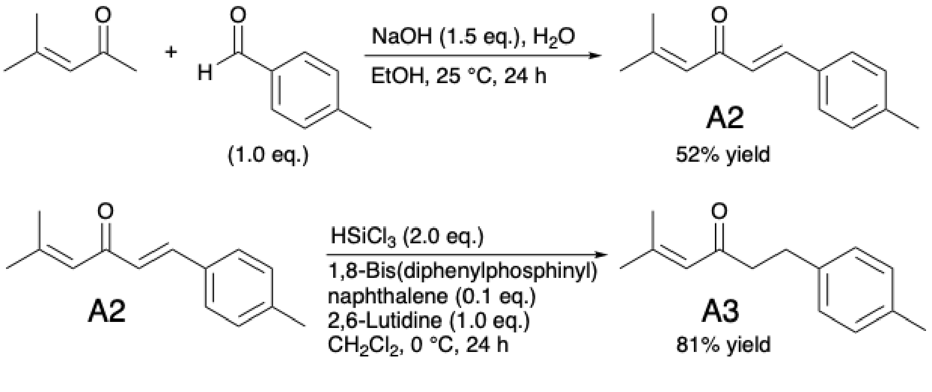

Figure S1. Schematic illustration of synthetic procedures of analog 2 (A2) and analog 3 (A3).

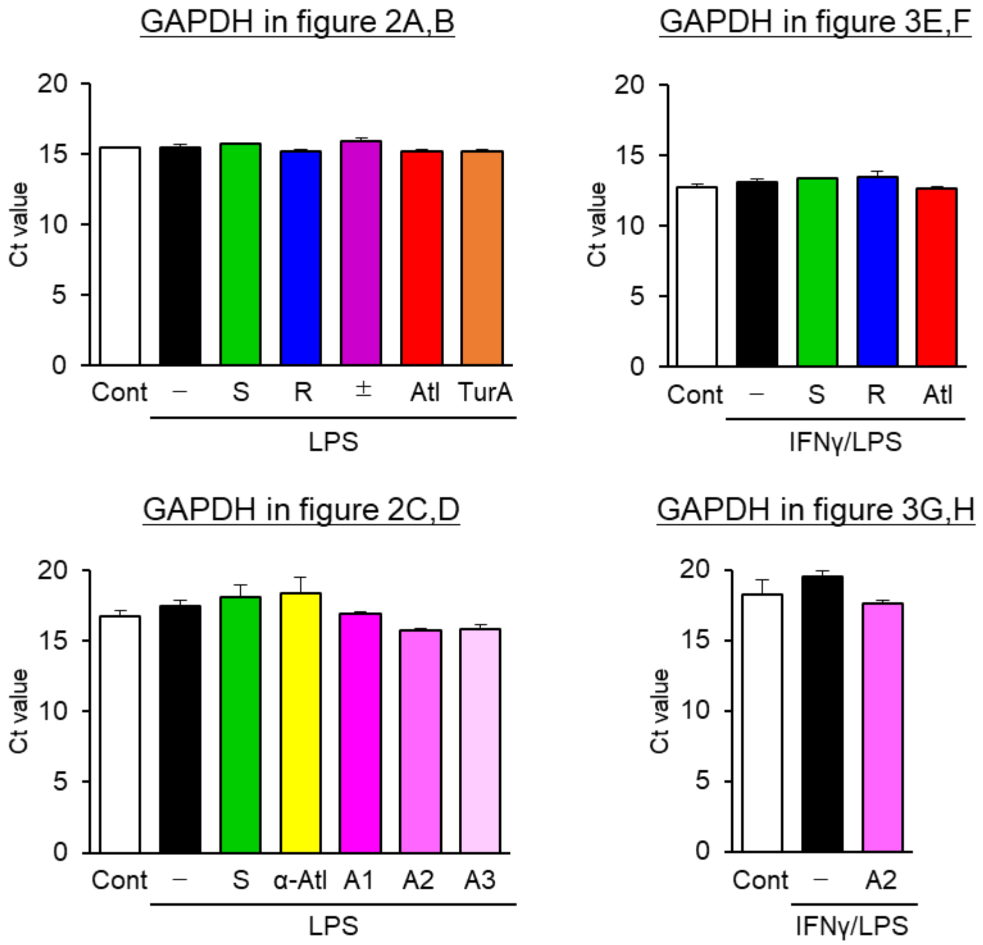

Figure S2. Ct values of GAPDH in all RT-qPCR experiments. Data are presented as the mean ± SEM of three independent samples. There were no significant differences between any two groups in each experiment ($n = 3$, one-way ANOVA, followed by a post hoc Tukey test).

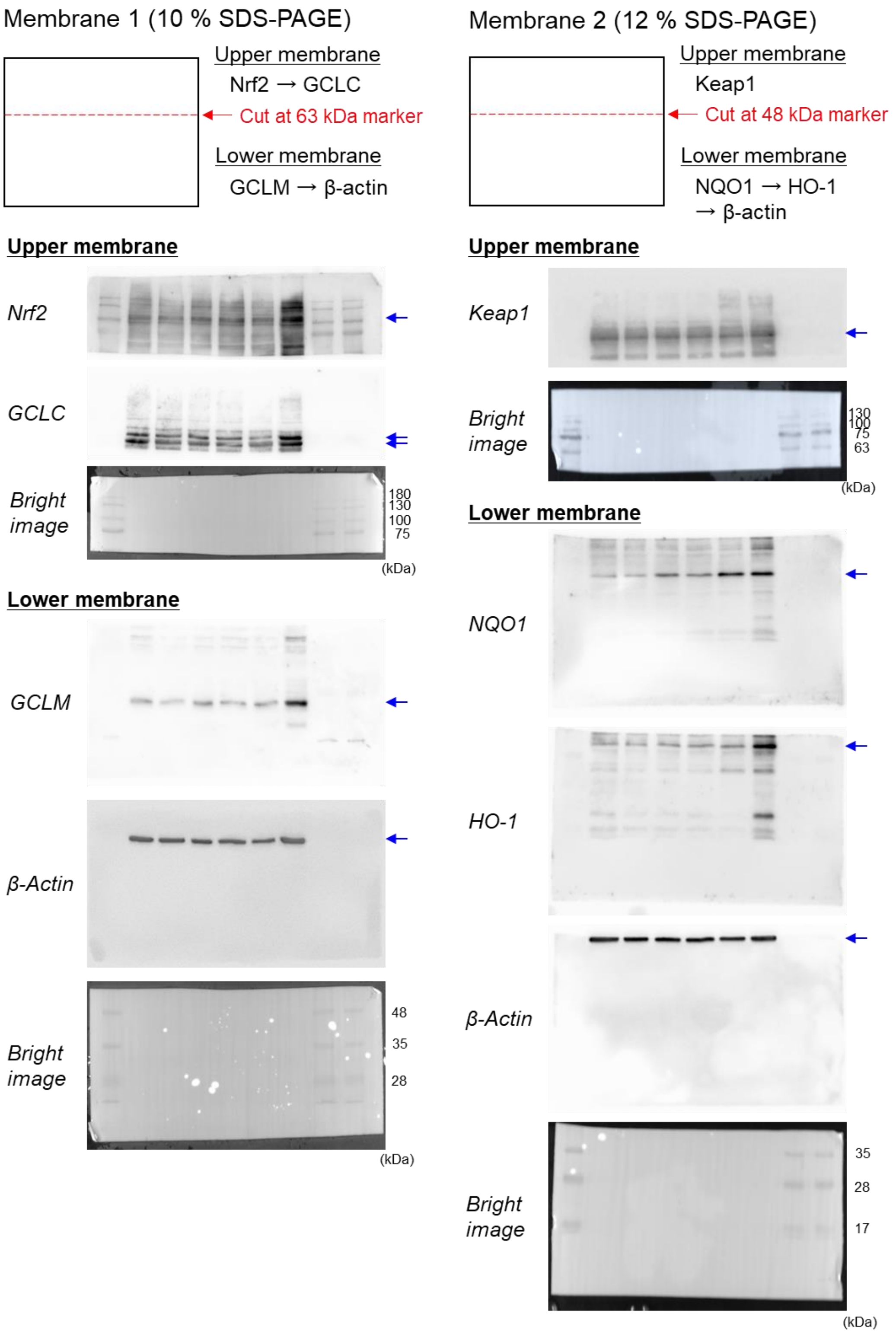

Figure S3. Whole images of immunoblot experiments in figure 6. The same samples were subjected to SDS-PAGE with 10% and 12% acrylamide gels. After transfer to PVDF membranes, both membranes were horizontally cut into two pieces at the position shown in red arrows. These divided membranes were blotted with different antibodies. Blue arrows indicate the major bands shown and analyzed in figure 6. Bright images indicate the images of blots with prestained molecular markers. Numbers beside the bright images indicate the sizes of marker bands.

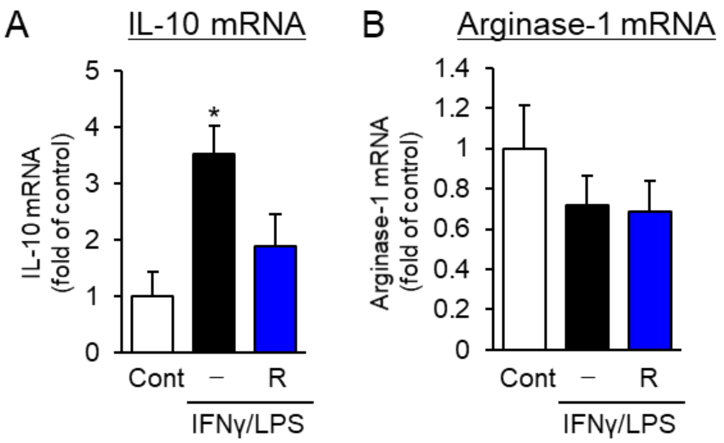

Figure S4. The effect of R-Tur on the markers of anti-inflammatory activation of microglia in the midbrain slice cultures. mRNA levels of anti-inflammatory activation markers (interleukin-10 (IL-10, A) and arginase-1 (B) in cultured slices were quantified by RT-qPCR. Data are presented as the mean ± SEM of 3 independent samples. * $p < 0.05$ vs. control (n = 3, One-way ANOVA, followed by a post hoc Tukey test).

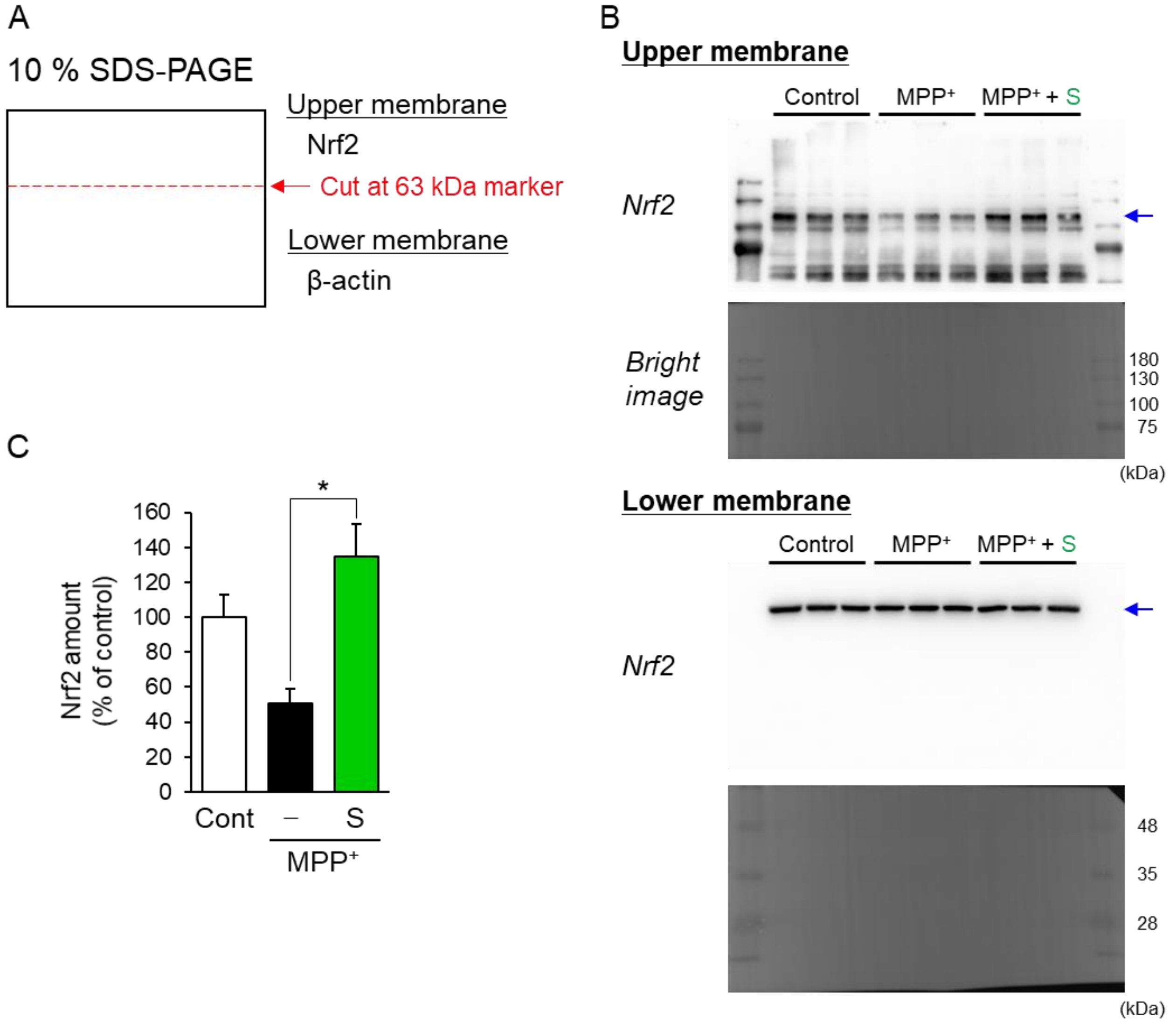

Figure S5. Immunoblot analyses of Nrf2 in LUHMES cells under control condition, treated with MPP^+ (10 μM), and cotreated with MPP^+ and S-Tur (20 μM) for 8 h. (A) Illustration of the scission of the immunoblot membrane. Protein samples were subjected to SDS-PAGE with a 10% acrylamide gel, followed by the transfer to a PVDF membrane. (B) Whole immunoblot images of upper and lower membrane, which were detected with anti-Nrf2 and anti-β-actin antibodies, respectively. Blue arrows indicate the bands that we quantified. Bright images indicate the images of blots with prestained molecular markers. Numbers beside the bright images indicate the sizes of marker bands. (C) Quantitative analysis of immunoreactive bands of Nrf2. The band intensity of each sample was normalized with the immunoreactivity of β-actin as an internal control. Data are presented as the mean ± SEM of three independent samples. * $p < 0.05$ vs. control (n = 3, one-way ANOVA, followed by a post hoc Tukey test).
